# Using Presurgical Biopsychosocial Features to Develop an Advanced Clinical Decision-Making Support Tool for Predicting Recovery Trajectories in Patients Undergoing Total Knee Arthroplasty: Protocol for a Prospective Observational Study

**DOI:** 10.2196/48801

**Published:** 2023-08-09

**Authors:** Karen Ribbons, Sarah Johnson, Elizabeth Ditton, Adrian Wills, Gillian Mason, Traci Flynn, Jodie Cochrane, Michael Pollack, Frederick Rohan Walker, Michael Nilsson

**Affiliations:** 1 Centre for Rehab Innovations University of Newcastle New Lambton Heights Australia; 2 Hunter Medical Research Institute Kookaburra Circuit New Lambton Heights Australia; 3 College of Health, Medicine and Wellbeing University of Newcastle Callaghan Australia; 4 College of Science and Engineering University of Newcastle Callaghan Australia; 5 College of Human and Social Futures University of Newcastle Callaghan Australia; 6 Hunter New England Local Health District Rankin Park Centre New Lambton Heights Australia; 7 Lee Kong Chian School of Medicine Nanyang Technological University Singapore Singapore

**Keywords:** biopsychosocial, total knee arthroplasty, prospective, recovery trajectories, patient-reported outcomes, predictive clinical decision tool, clinical decision support, knee arthroplasty, rehabilitation, psychosocial, patient-reported outcome, quality of life, patient recruitment, presurgery, patient stratification

## Abstract

**Background:**

Following total knee arthroplasty (TKA), 10% to 20% of patients report dissatisfaction with procedural outcomes. There is growing recognition that postsurgical satisfaction is shaped not only by the quality of surgery but also by psychological and social factors. Surprisingly, information on the psychological and social determinants of surgical outcomes is rarely collected before surgery. A comprehensive collection of biopsychosocial information could assist clinicians in making recommendations in relation to rehabilitation, particularly if there is robust evidence to support the ability of presurgical constructs to predict postsurgical outcomes. Clinical decision support tools can help identify factors influencing patient outcomes and support the provision of interventions or services that can be tailored to meet individuals’ needs. However, despite their potential clinical benefit, the application of such tools remains limited.

**Objective:**

This study aims to develop a clinical decision tool that will assist with patient stratification and more precisely targeted clinical decision-making regarding prehabilitation and rehabilitation for TKA, based on the identified individual biopsychosocial needs.

**Methods:**

In this prospective observational study, all participants provided written or electronic consent before study commencement. Patient-completed questionnaires captured information related to a broad range of biopsychosocial parameters during the month preceding TKA. These included demographic factors (sex, age, and rurality), psychological factors (mood status, pain catastrophizing, resilience, and committed action), quality of life, social support, lifestyle factors, and knee symptoms. Physical measures assessing mobility, balance, and functional lower body strength were performed via video calls with patients in their home. Information related to preexisting health issues and concomitant medications was derived from hospital medical records. Patient recovery outcomes were assessed 3 months after the surgical procedure and included quality of life, patient-reported knee symptoms, satisfaction with the surgical procedure, and mood status. Machine learning data analysis techniques will be applied to determine which presurgery parameters have the strongest power for predicting patient recovery following total knee replacement. On the basis of these analyses, a predictive model will be developed. Predictive models will undergo internal validation, and Bayesian analysis will be applied to provide additional metrics regarding prediction accuracy.

**Results:**

Patient recruitment and data collection commenced in November 2019 and was completed in June 2022. A total of 1050 patients who underwent TKA were enrolled in this study.

**Conclusions:**

Our findings will facilitate the development of the first comprehensive biopsychosocial prediction tool, which has the potential to objectively predict a patient’s individual recovery outcomes following TKA once selected by an orthopedic surgeon to undergo TKA. If successful, the tool could also inform the evolution rehabilitation services, such that factors in addition to physical performance can be addressed and have the potential to further enhance patient recovery and satisfaction.

**International Registered Report Identifier (IRRID):**

DERR1-10.2196/48801

## Introduction

### Background

Total knee arthroplasty (TKA) is a well-established and effective treatment for end-stage osteoarthritis [1]. The number of TKA procedures performed is increasing annually, both locally and globally. In both Australia [2] and the United States [3], it is projected that the number of TKAs undertaken will rise by 276% and 181%, respectively, by 2030, placing an increased burden on health care services. Surgical success rates are high and are currently based on a range of objective medical outcomes including survival, prosthesis performance, and revision rates [4]. However, despite this, there are consistent reports that up to 20% of patients report dissatisfaction with the outcomes of the procedure, reflected by enduring pain, limited function, and diminished quality of life [5-7].

Rehabilitation is an important factor that improves recovery from this surgery. However, no clinical guidelines are currently available to inform the recovery pathways. This is because there is no evidence to suggest who may benefit from rehabilitation, when and where this service should be delivered, and what services should be provided. Consequently, some patients receive inpatient (7-10 days) rehabilitation or at home rehabilitation through exercise monitoring (up to 10 weeks), whereas others are not referred to any form of rehabilitation, depending on the surgeon and the clinic. This inconsistent approach to rehabilitation may be because of the general lack of data demonstrating the effectiveness of any specific pathway. For example, a recent study demonstrated no difference in patient mobility at 26 weeks after the surgery when comparing inpatient rehabilitation with home-based monitoring [8]. Furthermore, a systematic review concluded that the setting did not significantly influence the outcomes [9].

During rehabilitation programs, the primary emphasis is on assisting patients in regaining muscle strength, enhancing joint stability, improving range of motion, restoring neuromuscular function, and recovering proprioception [10]. The approaches taken and outcomes vary widely among therapists, and to date, no clear guidelines exist for the most effective therapeutic strategies to be applied [9]. In addition, several psychological factors have also been reported to be linked to recovery following knee replacement or have the potential to impact recovery [11,12]. This includes psychological “vulnerability factors,” such as depression, anxiety [13-15], and pain catastrophizing [16,17], and “protective factors,” including resilience and committed action. However, despite their potential impact on recovery, quality of life, and satisfaction [12], these factors are not routinely reviewed in patients before surgery and remain overlooked in rehabilitation referral and ongoing clinical decision-making.

Determining whether rehabilitation programs are effective is also complicated by the use of inconsistent measures of “success.” Many studies to date have used a single traditional outcome measure, such as a physician’s or patient’s report of functional mobility, pain, or quality of life [18]. Although surgical success has been characterized by a number of objective medical outcomes, including survival, prosthesis performance, and revision rates [4], there has been increasing interest in considering and combining longer-term outcomes of success, such as patient satisfaction and social integration, with these other traditional measures. Over the last decade, the integration of patient-reported outcome measures (PROMs) in the evaluation of the success of TKA has become more widespread [19]. However, the impact of PROMs on clinical practices remains limited. Although there are clear benefits of assessing the patient’s perspective of recovery, including their symptoms, functional status, and quality of life [20,21], there remains a lack of consensus regarding the appropriate measures to be used, together with concerns of the cost-effectiveness involved in their application [22].

Regardless of the adopted outcome definition, no consistent set of factors that predict a successful surgery outcome have been identified. Individual studies have identified some factors associated with long-term outcomes. These include BMI, radiological arthritis severity (Kellgren-Lawrence score), mood status (eg, anxiety and depression), quality of life, and functional mobility [18,23,24]. Nevertheless, it is important to note that the collective predictive value of these metrics has not been externally validated, nor has their generalizability been demonstrated across various settings and populations [25].

Mounting evidence suggests that it may be possible to predict, via the use of a model, which patients could benefit from a more multifaceted approach to rehabilitation, which could encompass a broad range of biopsychosocial parameters, and how this can be precisely tailored to address each patient’s individual needs [26]. Importantly, if such a prediction model can be developed and validated, it would be extremely useful to guide the future provision of rehabilitation services to support superior outcomes for each patient. In a recent systematic review, we demonstrated that the number of clinically meaningful, well-validated prediction tools for recovery following total knee replacement is limited [26]. Several clinical and lifestyle factors have been linked to poor outcomes and dissatisfaction following a variety of surgical procedures, including TKA, such as female sex, obesity [27,28], lower social support level [29], and comorbidity status [15]. Indeed, predictive models have demonstrated that age and sex can predict improvement in knee function [30] and satisfaction [31]. However, few studies have reported the inclusion of psychological parameters in their model development. Moreover, many of the models developed have limited predictive success and lack external validation, which limits their implementation in routine clinical use. There are also constraints when predictive models repurpose existing data sets owing to the limitations of data quality and completeness [32].

To date, there is an unmet need for the development of a clinical tool based on a comprehensive range of parameters that can assist in determining individual recovery trajectories following TKA, which can be easily adopted in the clinical setting. Through the identification of factors influencing patient recovery trajectories across a range of success outcomes, rehabilitation pathways will be better informed regarding the tailoring of services to meet patients’ individual needs. This has the potential to improve long-term outcomes and satisfaction by addressing a broader range of pre- and postoperative parameters.

### Aims and Objectives

The major aim of this study is to develop a clinical tool that can be used in the prediction of recovery trajectories, which can inform rehabilitative services regarding the nature of potential interventions and the timely delivery of the right services to each individual patient. Our approach will be to look beyond the impact of biomedical features alone on patient outcomes by adopting a biopsychosocial approach. The biopsychosocial model of health systematically considers biological, psychological, and socioenvironmental factors and their complex interactions in understanding illness, health, and health care delivery. It encompasses a more holistic approach to understanding illness and wellness than a purely biological vision. The tool will be constructed by assessing the predictive capacity of a comprehensive battery of biopsychosocial factors on a number of patient outcomes following TKA, including improvements in knee symptomology, quality of life, and satisfaction. This approach has the potential to facilitate the allocation of rehabilitation resources toward a more holistic approach to recovery following surgery and could improve the effectiveness of TKA across a range of success outcomes, including patient satisfaction. By supporting the delivery of rehabilitative interventions that are precisely targeted to patient needs, this clinical decision-making tool could also improve the efficiency of health care resource delivery.

## Methods

### Study Setting

This study was undertaken with patients who underwent TKA surgery between November 2019 and June 2022 across 4 participating Ramsay Health Care facilities in New South Wales (NSW), Australia, including Lake Macquarie Private Hospital, Gateshead; Kareena Hospital, Caringbah; Baringa Hospital, Coffs Harbour; and Wollongong Private Hospital, Wollongong. Surgical procedures were performed by 11 collaborating orthopedic surgeons.

### Study Design

**Figure 1:**
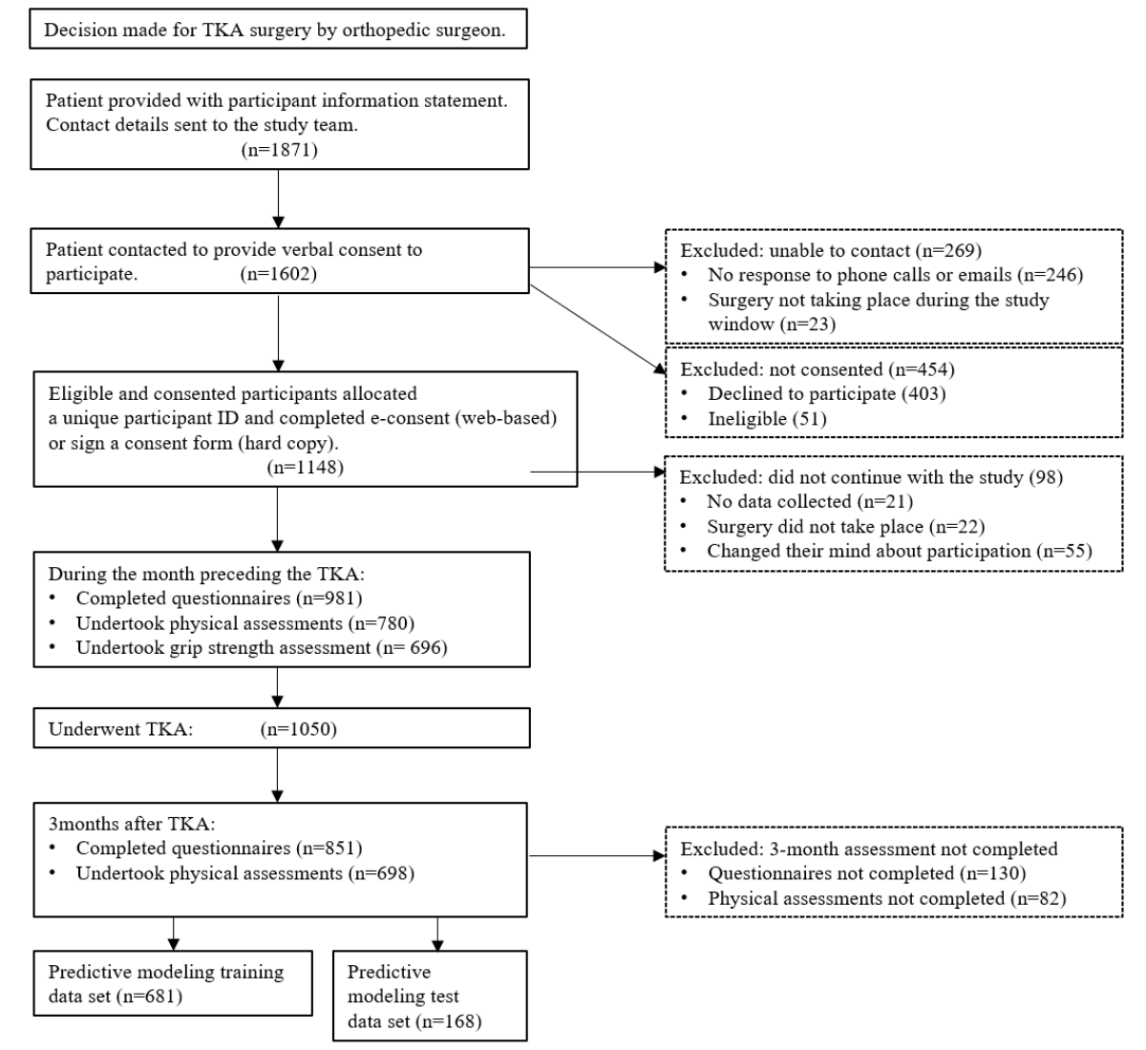
Participant timeline. TKA: total knee arthroplasty.

This study had a prospective observational design. Participants were involved in the study for a duration of 4 months, with data collection taking place at 2 time points during this period: within the month preceding their scheduled TKA and 3 months (12 weeks) following the TKA procedure. Figure 1 outlines the participants’ timeline for this study and the number of participants recruited and excluded at each stage.

### Study Population and Eligibility

All patients identified by the participating orthopedic surgeons as requiring a total knee replacement and who fulfilled the eligibility criteria were invited to participate in the study. Patients were included as eligible if they were aged ≥18 years, could complete the PROMs questionnaires, and confirmed to require a primary total knee replacement (bilateral or unilateral procedure). Patients were excluded from participating if they were receiving a primary total knee replacement owing to trauma, had undergone previous knee surgery within the past 6 months, were planning to undergo additional knee surgery within the next 12 months, or did not have the capacity to provide informed consent to the research.

### Sample Size

For machine learning modeling, the standard required sample size used should ensure at least 10 events for each included predictor parameter [33]. In this study, we have a total of 39 input variables and 76 input events when applying the conversion of categorical variables to numerical data (one-hot-encoding). Applying the standard approach, we would require 390 participants for models without one-hot encoding and 760 participants when using one-hot encoding.

Recently, Riley et al [34] have developed a more refined calculation of the required sample size, which depends not only on the number of candidate predictor parameters but also on the total number of participants, the outcome proportion (incidence) in the study population, and the expected predictive performance of the model. Applying the method proposed by Riley et al [34] to our classification modeling with an aim of 90% accuracy and an outcome proportion of 20% (ie, 20% of participants were not expected to meet the minimal clinically important difference [MCID] improvement threshold), a predicted *R*^2^ of 0.5, and shrinkage of 0.85, returns a required sample size of 864.

In this study, we aimed to recruit at least 1000 patients undergoing total knee replacement. This will ensure sufficient data for inclusion in our machine learning predictive modeling analyses and allow for 13.6% (136/1000) of data to be nonevaluable owing to missing data fields.

### Recruitment and Enrollment Procedures

Eligible participants were contacted by the study team through a follow-up phone call made after they received the study Patient Information Sheet from their surgeon. Eligible participants who chose not to participate when they received the Patient Information Sheet from their surgeon were not contacted. If the participant verbally agreed to participate in the study, they were provided access to study materials either on the web or by post, and if they consented to undertaking physical assessments of mobility via a video call, a time for this appointment was made at a time convenient to the participant.

If patients opted to have their study questionnaires accessed via the study-specific web-based platform, they were provided with a copy of the Patient Information Sheet when they accessed this system via a participant-specific log-in. Following a review of the Patient Information Sheet, they were asked to acknowledge their consent to participate in the study and provide electronic consent on the Electronic Letter of Consent Form. Questionnaire templates were not accessible to the participants until the consent field on this form had been completed. Participants also had the option to consent to all study assessments being undertaken or to only complete the study questionnaires.

If patients opted to have their study questionnaires posted to them, a copy of the Patient Information Sheet and Letter of Consent were included in the posted package. The participants were asked to sign the consent form before completing the questionnaires. The completed consent form and questionnaires were subsequently posted back to the study team.

Participation in this study was voluntary. Patients had the option to decline participation at any time point during the study and were informed that this would not impact their ongoing medical care. If patients decided to withdraw from the study, they could specify whether their data were to be removed from the study or if they were willing for it to be used as part of the analysis.

### Data Collection Tools and Procedures

#### Web-Based Data Collection Platform and Centralized Data Repository

Data were entered and stored on the secure electronic platform Vision Tree Optimal Care (VTOC) developed by VisionTree Software, Inc. This platform enables the direct entry of data by participants when completing study documents and serves as a centralized data repository. The addition of an e-consent form, as the first item viewed and completed by participants when accessing the study-specific platform, enabled consent to be obtained before the collection of study data. Participants accessed the site via a unique encrypted link sent to their email. Using the encrypted link, the participants also validated their identity by entering their initials on the platform.

The electronic site is 256-bit Secure Sockets Layer (SSL) encrypted to protect the privacy of the participants. The VTOC meets all Health Insurance Portability and Accountability act of 1996 security and compliance standards, which include physical, technical, and procedural safeguards to help protect personal information. Using Thawte as a third-party encryption service, all reminders or messaging are 256-bit SSL encrypted.

The participant log-in links are encrypted using Advanced Encryption Standard-256 encryption. The participant internal identification number, only known to the VTOC application, is included in the encrypted payload, thus ensuring that it is unique to the participant. Advanced Encryption Standard is a symmetrical-key algorithm, meaning the same key is used for both encrypting and decrypting the data.

The platform can be viewed by any modern browser, whether a tablet, mobile, or computer-based system. No data were housed on the device at any point; therefore, if a device gets lost, there was no exposure of any of the data within the platform or data being collected.

VisionTree’s security technologies include an F5 Load Balancer and Cisco Firewall. Software technologies include 256-bit SSL, third-party verification, command line and basic virus scanning, audit logs, and secured biometric access to the data centers. The VTOC platform firewall was OSCI level 4 compliant.

#### Patient Deidentification and Study Code Allocation

Unique study code or ID numbers were automatically and randomly assigned to each participant within the VTOC. Reports were available from the system using deidentified data, that is, study code or ID numbers. Members of the study team involved in data management, processing, and analysis only have access to the data that have been deidentified. Identifiable data can be accessed by study team members who need to make direct contact with participants during study visits or for the entry of additional data.

#### Study Questionnaires

##### Overview

Consented participants were asked to complete the study questionnaires either on the web using the VTOC platform or on a posted hard copy. Questionnaires were disseminated for completion at 2 distinct time points: within 4 weeks before their scheduled TKA and at 3 months after their TKA. These questionnaires are validated self-report tools that are used to measure a variety of biopsychosocial parameters associated with recovery following TKA.

Biopsychosocial measures and self-report tools used to derive each measure and administration time points are shown in Table 1. Psychosocial variables were selected following a thorough literature review that was conducted to determine the types of constructs that had demonstrated associations with outcomes that were relevant to the outcomes of interest in this study. Many of the selected variables were supported by systematic review–level and meta-analytic–level evidence. In addition, because of the innovative nature of the proposed tool, we also wanted to explore the role of other factors that had good face validity with respect to their potential to enhance or hinder someone’s behavioral engagement in rehabilitative activities or general restoration of functioning [12]. Measurement tools were selected based on their psychometric properties and length (shorter scales were selected where possible to reduce the strain on participants).

Participants were also asked before their TKA if they had experienced any falls or unexpected weight loss within the last 12 months. In addition to measuring biopsychosocial parameters before surgery, participants were asked about their expectations about surgical outcomes, enabling them to perform specific activities, as well as their expectation of experiencing a reduction in knee pain.

**Table 1:** Biopsychosocial measures and administration time points.

Measure	Tools	Before TKA^a^	3 months after TKA
Knee symptomology	Western Ontario and McMaster University Osteoarthritis Index [35]	✓	✓
Mood status (depression, anxiety, and stress)	Depression Anxiety and Stress Scales-21 [36]	✓	✓
Pain catastrophizing	Pain Catastrophizing Scale [37]	✓	
Resilience	Brief Resilience Scale [38]	✓	
Committed action	Committed Action Questionnaire-8 [39]	✓	
Capacity to succeed in achieving goals	Valued Living Scale [40]		✓
Quality of life	Short Form-12 version 2 [41]	✓	✓
Social support	Medical Outcome Study Social Support Survey, 6 item [42]	✓	
Employment status	—^b^	✓	
Physical activity	University of California, Los Angeles Activity Scale [43]	✓	✓
Sleep quality	Extract from the Pittsburgh Sleep Quality Index [44]	✓	✓
Nutrition status	Starting the Conversation tool [45]	✓	
Alcohol consumption	The Alcohol Use Disorders Identification Test-Consumption [46]	✓	
Smoking behavior	—^b^	✓	✓
Concomitant medical conditions	Charlson Comorbidity Index [47]	✓	✓

^a^TKA: total knee arthroplasty.

^b^As reported by the participant.

##### Knee Symptomology

We implemented the Western Ontario and McMaster University Osteoarthritis Index (WOMAC) [35] to evaluate the level of knee function limitation, pain, and stiffness symptom severity. The WOMAC is a validated and widely used self-administered questionnaire [48] developed to provide a means of standardizing self-report health status and activities relevant to patients. It consists of 24 items: 5 relating to pain, 2 to stiffness, and 17 function items with ratings provided using a 5-point Likert scale. Lower scores are indicative of lesser symptoms, and higher scores are indicative of worse symptomology. The global score is the sum of the scores for all the items combined. In this study, participants were asked to complete the WOMAC before their TKA and at 3 months after their surgery.

##### Mood Status

The mood status of the study participants was assessed using the Depression Anxiety Stress Scales (DASS)–21 [36]. Higher scores are indicative of higher levels of depression, anxiety, and stress. All scores derived from the 21-point scale were multiplied by 2 to enable comparison with the full 42-point DASS and to determine clinical cut-offs for symptom severity. The full DASS and abbreviated DASS-21 have been validated to measure the 3 negative emotional states (ie, depression, anxiety, and stress) in research and clinical settings, but is not considered a diagnostic tool or a replacement for a comprehensive clinical interview [49]. Each of the DASS-21 subscales contains 7 items. The Depression scale assesses dysphoria, hopelessness, devaluation of life, self-deprecation, lack of interest or involvement, anhedonia, and inertia. The Anxiety scale assesses autonomic arousal, skeletal muscle effects, situational anxiety, and the subjective experience of anxious affect. The Stress scale is sensitive to levels of chronic nonspecific arousal. It assesses difficulty relaxing; nervous arousal; and being easily upset or agitated, irritable or overreactive, and impatient. Participants are asked to use a 4-point severity or frequency scales to rate the extent to which they have experienced each state over the past week. The scores for depression, anxiety, and stress are calculated by summing the scores for the relevant items. In this study, participants were asked to complete the DASS-21 before their TKA and at 3 months after their surgery.

##### Pain Catastrophizing

We used the Pain Catastrophizing Scale (PCS) [37] to measure the level of catastrophic thinking related to pain before TKA. PCS is one of the most widely used instruments for measuring pain catastrophizing and has been used extensively in clinical practice and research settings [50]. The self-report form consists of 13 items describing thoughts and feelings that patients may experience when they are in pain. Respondents provided ratings for each item on a 5-point Likert scale ranging from 0 (“not at all”) to 4 (“all the time”). Higher scores are associated with higher amounts of pain catastrophizing, with scores >30 associated with a high risk of chronic pain.

##### Resilience

We assessed participants’ resilience before their TKA using the Brief Resilience Scale (BRS) [38]. This scale assesses an individual’s ability to recover from stressful events (level of resilience), and has demonstrated relationships with positive postsurgical health, satisfaction, and pain outcomes [12]. The BRS contains 6 items, including 3 positively and 3 negatively worded statements (reverse scored). Respondents rated their responses using a 5-point Likert scale ranging from 0 (“strongly disagree”) to 4 (“strongly agree”) for each statement. Raw scores are calculated by summing the item scores and range from 6 to 30. The BRS-weighted score is calculated by dividing the summed item scores by the number of items and ranges from 0 to 5. Higher scores are indicative of greater resilience.

##### Committed Action

Committed action is a component of “psychological flexibility” and is defined as a person’s capacity to flexibly engage in effective and adaptive behaviors, guided by personally held values, even in the presence of challenges and discomfort [39,51]. We assessed committed action in our study cohort before their TKA because of the role this psychological process has been shown to play in an individual’s health outcomes and their management of pain [12]. We used a shortened form of the Committed Action Questionnaire-8 [39], which comprised 8 statements. Respondents rate the extent to which each item applies to them, ranging from 0 (“never true”) to 6 (“always true”). The total scores are calculated by summing the item scores, with a maximum score of 48. Higher scores denote greater committed action.

##### Capacity to Succeed in Achieving Goals

The Valued Living Scale assesses a person’s goal importance, success, and confidence with respect to 8 value domains [40]. The scale was used to assess a person’s rating of the importance of each of the 8 goals, their confidence in achieving these goals, and their success in achieving them following TKA.

##### Quality of Life

To evaluate participants’ quality of life before and after TKA, we used the Short Form-12 (SF-12, version 2) quality of life questionnaire [41]. This tool is a 12-item self-administered measure of general quality of life that is widely used and has been validated in an Australian population [52]. It is a shortened and modified version of the original 36-item form [53]. Scores are transformed to generate 2 weighted summary scores for physical health, PCS, and mental health, the Mental Component Scale. Scores range from 0 to 100, with higher scores indicating a better health state. In this study, the participants completed the SF-12 before their TKA and at 3 months after their TKA.

##### Social Support

The amount of social support available to patients has been reported to influence physical and mental well-being [54] as well as being a key determinant of the suitable location of postsurgical rehabilitation services (ie, inpatient vs community based). The level of social support that participants felt they had available to them before their TKA was assessed using the Medical Outcomes Study Social Support Survey, 6 item [42]. The survey captures information related to perceived psychological and material support derived from interpersonal relationships. Scores ranged from 6 to 30, with higher scores indicative of higher levels of perceived support.

##### Physical Activity

Recent activity levels of the participants were determined using the University of California, Los Angeles Activity Index, which was developed to assess activity levels in patients undergoing joint replacement [43]. Participants were asked to indicate which of the 10 activity levels provided best reflected their current level of activity. The activity levels included being inactive (1 and 2), undertaking mild activities (3 and 4), moderate activities (5 and 6), active events (7 and 8), and impact sports (9 and 10). Activity status was evaluated before the TKA and at 3 months after the TKA.

##### Sleep Quality

To assess sleep quality in our study cohort, we used the sleep quality question extracted from the Pittsburgh Sleep Quality Index [44]. Participants were asked to rate their current sleep quality on a Likert scale ranging from 1 (“very bad”) to 4 (“very good”) on 2 occasions: before their TKA and at 3 months after the TKA.

##### Nutrition Status

The dietary habits of participants before TKA were assessed using the Starting the Conversion Score [45]. The Starting the Conversion Score is a validated 8-point screening tool used to evaluate dietary habits. The participants rate the frequency of ingesting a range of 8 different food types over the past few months. The total scale ranges from 0 to 16, with higher scores associated with worse dietary habits.

##### Alcohol Consumption

Alcohol consumption before TKA was evaluated using a modified version of the Alcohol Use Disorders Identification Test [55] related to alcohol consumption only (Alcohol Use Disorders Identification Test-Consumption) [46]. Participants were asked 3 questions related to their average alcohol consumption over the last 12 months, with a maximum total scale score of 12. Total scores <5 indicated low-risk consumption and scores >5 indicated hazardous or harmful consumption.

##### Smoking Behavior

Before and 3 months after the TKA, we asked participants to indicate their smoking status. Smokers were asked to provide details regarding smoking frequency.

##### Concomitant Health Conditions

The Charlson Comorbidity Index provides a weighting to concomitant health conditions based on the nature of the condition and age of the individual and is used to predict 1-year mortality. The higher the score, the higher the mortality risk [56]. We applied the same 10 medical conditions and weightings used by Chaudry et al [47]. Participants were asked to indicate whether they experienced any of the 10 medical conditions provided before their TKA and at 3 months after TKA.

#### Outcome Recovery Measures—3 Months After TKA

Table 1 shows the PROMs questionnaires completed by the participants 3 months after TKA and the parameters measured. Questionnaires related to knee symptomology (WOMAC), quality of life (SF-12), mood status (DASS-21), and lifestyle (physical activity, sleep, and smoking behavior) were also repeated at the 3 months post-TKA time point. This allowed us to evaluate changes or improvements in these factors at 3 months after TKA compared with levels measured before surgery. The primary outcome measure in our study is the improvement in quality of life 3 months after TKA (changes in SF12 physical and mental health scores).

Secondary outcome measures include knee symptomology (changes in WOMAC scores), satisfaction with the surgery, and mood status (changes in DASS-21 scores) 3 months after TKA.

We also asked questions related to satisfaction in achieving specific activities, the level of knee pain reduction, and overall satisfaction with the surgery. Information related to the type of rehabilitation they undertook and health care services used following their surgery were also collected.

#### Study Participation Evaluation Survey

A study participation evaluation survey was included to enable the participants to provide the research team with feedback regarding the assessments they were asked to undertake and the overall conduct of the study. It also allowed the study cohort to share their suggestions for future research areas they considered important to be addressed in the recovery from TKA surgery. The results from this survey will inform our team’s future research endeavors in similar cohorts.

#### Physical Assessments

##### Overview

When participants were contacted by phone regarding their potential involvement in the study, they were also asked if they consented to undertake 2 physical tests in their home, taking 15 minutes, that enabled measurements of their mobility, balance, and strength. These assessments were undertaken via a video call between the participants in their home and a member of the study team in their office. To participate in these calls, the participant needed to have the following:

- Sufficient internet access and a mobile phone, tablet device, or computer with a movable camera
- Someone to be at home with them during the call, to assist with holding the device during the call and be there for safety support if the participant stumbles or falls during the assessments
- A dining chair or a similar chair with a firm seat and arm rests to perform assessments
- A 4-m length floor space with uniform flooring and no trip hazards to perform assessments

Physical assessments were conducted on 2 occasions during the study, within 2 weeks before their scheduled TKA and at 3 months after the TKA.

In our original protocol, we planned to conduct face-to-face physical assessments with participants at each of the 4 study sites. As these activities were restricted by the COVID-19 pandemic, we adopted the approach to undertake these measures via video calls with participants in their home. This approach enabled these assessments to be performed despite social distancing regulations being implemented at the time of the study. It also enabled the study to be managed from a central location and still involve participants located across a large geographical range.

The physical measures applied in this study were selected following a thorough literature review that was conducted to determine the types of assessments used to assess joint function, mobility, and knee; were extensively validated; and were used to assess improvements or changes associated with total knee replacement surgery. As these assessments needed to be undertaken via video calls between participants and study staff, they also needed to have the capacity to be accurately measured without supervision by a physiotherapist and, if possible, not require specialized equipment.

##### Timed Up and Go Test

The Timed Up and Go test is a simple, reliable, and valid test of functional mobility that can be used to monitor clinical changes over time [57]. It enabled an evaluation of the participants’ capacity to engage in simple daily activities involving walking and getting up from a chair. We performed the Timed Up and Go test before the TKA and at 3 months after the TKA. On each occasion, the test was performed twice, with the fastest time for each individual to get up from a chair, walk to a 3-m mark, turn around, and come back and sit down recorded. Therefore, the shorter the time taken to perform the test, the better the performance.

##### 30-Second Sit-to-Stand Test

The 30-second Sit-to-Stand (30 s-STS) test is a reliable and valid measure of lower extremity strength [58]. We performed the Sit-to-Stand test on 2 occasions, before the TKA and 3 months after the TKA. On each occasion, the total number of completed chair stands achieved over a 30-second period was recorded. A larger number of stands is indicative of better performance. The test is a general measure of the strength and endurance of a person’s legs and is sensitive to change when a person’s knee pain and function changes.

##### Presurgery Hand Grip Strength Assessment

Hand grip strength measures the maximum amount of static force that a person can apply by their hand while gripping a handheld dynamometer. It is a reliable proxy for overall strength and is related to the risk of frailty, falls, functional capacity, morbidity, and mortality [59,60]. Hand grip strength was assessed before the surgery. As this assessment requires the use of a calibrated dynamometer (Jamar Plus+ Hand Dynamometer, Jamar Diagnostics), a device was provided to each study site. The hospital site staff received training from a member of the research team before the start of the study to conduct these assessments.

#### Demographic and Clinical Information

To reduce the data collection strain on study participants, we extracted demographic data from patient hospital records at each study site. In addition, medical data related to knee pathology diagnosis and medications were also collected from the hospital records. The scope of additional patient data used in this study is shown in Textbox 1.

**Demographic and medical data derived from patient hospital records.**

**Demographic and lifestyle behavior**

- Age at the time of surgery
- Sex
- Nationality
- Suburb or postcode (rurality)
- Working status

**Medical**

- BMI
- American Society of Anesthesiologists score
- Surgery type (unilateral or simultaneous bilateral)
- Diagnosis
- Concomitant medications (were >5 different medications taken before surgery? What type of medications were taken before surgery?)
- Anesthetic and analgesic agents
- Length of hospital stay

### Data Analysis

#### Overview

For prediction modeling, we are interested in whether a combination of presurgery measures can predict the change in physical (WOMAC subscores) and quality of life (SF-12 subscores) for participants at 3 months after surgery. Both the absolute improvement and whether a patient reported an MCID in improvement between the preoperative and postoperative stages will be predicted, requiring the implementation of regression and classification prediction models, respectively. The distribution-based MCID for each outcome measure will be determined according to the method outlined by Carender et al [61]. Every measure conducted before the surgery, including those from PROMs, physical measures, and medical records, will be considered as potential inputs (predictors) for the model. The data will be divided into 2 sets: a training set, consisting of 80% (840/1050) of the cohort chosen at random, and a test set, consisting of the remaining 20% (210/1050) of the cohort.

#### Data Cleaning

Although the web-based forms required each question set to be answered completely before the next was commenced, some participants used paper-based forms, and questions may have been missed. Moreover, some participants were unable to attend the video calls for the measurement of physical parameters, and in some cases, their medical records were incomplete. Any inputs with low variance or with >30% missing data will be discarded from the analysis. To deal with missing information in the remaining inputs, we will use multiple imputation, whereby missing measurements are replaced with the population mean (or the population mode for categorical measures). Imputation will be conducted independently on the training set without reference to the test set.

#### Models

We will consider standard linear models for prediction (ie, multivariable linear regression) with and without regularization and will also consider nonlinear models (decision trees, random forest, gradient boosting, neural networks, and Bayesian soft decision trees). Models will be trained both with and without one-hot encoding for the categorical variables.

#### Training

Models will be trained using 5-fold cross validation on the training set. The metric used to select the best classification model will be accuracy (ie, the percentage of patients classified correctly).

For each model, we will use a grid search for hyperparameter optimization. For example, a decision tree model will be trained for a range of depths and a range of minimum number of samples required at the leaf nodes, and the combination that returns the best model will be the chosen hyperparameters. We will also consider the subset of inputs used to train a model as a hyperparameter and will use exhaustive search to find the best input subset (ie, to find the best model that uses only 7 predictors; every possible combination of the 7 predictors will be tested). This avoids training a model using a “greedy” algorithm, which is not guaranteed to find the best combination of inputs.

#### Validation

The performance results of the chosen models will be validated on the test set containing the remaining 20% (210/1050) of the cohort. The root mean square error, *R*^2^, and error rate will be reported on the training set, and plots showing the predicted versus measured outputs will be included in the supplementary materials in future papers. Where the model type allows it, we will also report CIs for the parameters and predictions.

We will then apply a common post hoc method to establish if the sample size is sufficient for the reported error rate for each model as follows: taking an increasingly large subset of the data, we will train the model, calculate the error, and plot the error as a function of the number of observations in the subsample. Fitting a power law curve, will establish an estimate of the sufficient sample size. Where the model type allows it, we will also report CIs for the parameters and predictions.

All machine learning methods will be conducted using the Python programming language. The *pandas* and *numpy* packages will be used for data cleaning, with the *scikit-learn* package used for training with standard machine learning models and techniques, and custom Python code will be used for training the Bayesian soft decision trees.

### Ethics Approval

This research methodology was peer-reviewed and approved by the School of Medicine and Public Health at the University of Newcastle, NSW, Australia, in accordance with the Australian Code for the Responsible Conduct of Research. This study was conducted in accordance with the National Statement on Ethical Conduction in Human Research (2007). Ethics approval was granted by the University of Newcastle Human Research Ethics Committee (approval H-2019-0109) on June 21, 2019. Approval for each study site’s participation was reviewed and approved by the Ramsay Research and Governance Office in NSW, Australia (RHC RG-2019.008).

## Results

This study was funded by the Ramsay Hospital Research Foundation in September 2018. Recruitment for this study occurred between November 2019 and June 2022. Data collection was completed in September 2022. The study results will be available within 12 months of the final data collection on completion of the machine learning data processing of the predictive models (ie, final results are expected by September 2023).

During the recruitment phase, 1871 individuals requiring TKA were identified by the collaborating orthopedic surgeons across all 4 study sites, with a total of 1050 eligible, consented patients undertaking at least 1 of the pre-TKA study assessments in the study cohort (Figure 1). Participants for whom a complete data set was collected (ie, responses to all study questionnaires at both time points) were included in the machine learning data processing for predictive model development (n=851). Participants were randomly allocated for analysis into 2 groups, such that 80% (681/851) were included in the model training data set and 20% (170/851) were included in the model test data set.

## Discussion

### Expected Findings

This study responds to an unmet clinical need to address factors that can improve outcomes in patients undergoing total knee replacement. With a substantial increase in the number of TKA procedures expected to take place over the next decade, there is the potential for a significant number of patients to be dissatisfied with their surgical outcomes, unless the issue can be effectively addressed.

We anticipate the development of a clinical predictive tool that has the potential to improve patient-centered TKA outcomes. The uniqueness of the model being used is the inclusion of a comprehensive assessment of modifiable psychological, resilience, and clinical factors that have been linked to TKA recovery outcomes.

### Comparisons With Prior Work

The approach adopted in this protocol builds on existing research demonstrating the involvement of factors influencing recovery of patients following total knee replacement and the inclusion of these factors in predictive modeling of postsurgery outcomes. We are unaware of any published predictive models of patient outcomes following TKA that include such a comprehensive battery of biopsychosocial parameters as those being evaluated in our study protocol. Our systematic review of predictive models for TKA outcomes [26] and those undertaken by others [32] revealed that the most consistently used presurgical parameters included clinical and medical factors such as age, sex, BMI, and concomitant health conditions, whereas psychological factors were incorporated to a lesser extent and, when applied, were restricted to measures of mood status (depression and anxiety) or mental health quality of life. There have been several specific modifiable psychological factors, including pain catastrophizing [18,62] and resilience [63], which have been associated with recovery following TKA and could inform the optimization of patient outcomes. However, their predictive capacity for patient outcomes after TKA has not been widely explored.

Previous limitations of predictive models are associated with using data derived from existing registries or retrospectively collected data sources [26]. Such approaches can be problematic because of the need for data imputation caused by missing values or limitations in predicting specific outcomes owing to insufficient presurgery variables collected. The prospective collection of specific variables of interest, as we propose in this study, enables the collection of specific input data that have been associated with specific outcomes. This approach has the potential to inform appropriate rehabilitation interventions to improve patient outcomes.

### Potential Impact and Clinical Implementation

If successful, this project will provide a tool that can help guide health professionals with respect to comprehensive rehabilitation decisions individualized to each patient. The tool will underpin and support the identification of biopsychosocial risk factors for poor postoperative holistic outcomes and facilitate the development of biopsychosocial prehabilitative and rehabilitative interventions, which could lead to improved overall outcomes. In addition, the tool will enable efficient patient stratification according to identified individual needs, which allows a vastly more targeted service provision, maximizing the potential for impact. Currently, patients are infrequently evaluated using formal, standardized assessments for the type of rehabilitative service they would most likely need and benefit from. This project will provide a means of stratification of patients in a clinical setting, identification of “at risk” individuals early in their recovery journey, and support appropriate follow-up services to be implemented.

The scope of the factors influencing decision-making will be evolved. Typically, when patient information is collected, it generally focuses on selected medical or targeted joint parameters [64] and does not take consider parameters such as patient anxiety, stress resilience, quality of pain, social support, and sense of purpose, all of which have recently been shown to be associated with patient outcomes [12]. The model used in this study will explore the influence of these additional parameters on recovery and well-being following TKA, thereby providing evidence for a more comprehensive therapeutic strategy.

### Strengths and Limitations

In this study, we are collecting data prospectively, which enables the scope of features that may impact patient outcomes to be extended beyond that routinely collected in existing data sets and provide a focus on the biopsychosocial parameters of interest. The development of a clinical decision tool that is focused on a broader scope of patient features affords the opportunity for a more holistic approach to be taken in rehabilitation programs that extend beyond physical recovery strategies alone. The design of the study has attempted to reduce surgeon and hospital bias by including patients who underwent TKA by 11 different orthopedic surgeons across 4 different hospitals. These facilities were located across a large geographical range and enabled the inclusion of participants residing in metropolitan and rural communities to take part in the study. This was further enhanced by the capacity to collect data on the web and via video calls, which enabled the study to be managed at a central location and include participants across a large geographical area.

Although the planned study will evaluate the predictive capacity of a comprehensive battery of biopsychosocial parameters on TKA patient outcomes and offer significant contributions to the existing literature, there are potential limitations. In this study, we are assessing the predictive capacity of presurgical parameters on recovery outcomes assessed at 3 months after TKA. This may be considered early on in a patient’s recovery journey. It has been demonstrated that the maximal changes in patient-reported knee symptomology occur rapidly within the first 3 months, with subsequent improvements continuing, albeit at a slower rate, up to 12 months after surgery [65,66]. It is possible that the impact of psychological factors on patient outcomes may differ at later time points, as patients’ circumstances and needs change, and as such, predictive modeling developed in this study may be limited to early recovery outcomes.

We recognize that our study protocol does not encompass all factors that could potentially influence outcomes after TKA. Our analyses do not include surgical factors known to impact outcomes, including factors such as the surgeon or hospital, prosthesis type, and surgical procedures [67], and radiological factors, such as the Kellgren-Lawrence score [23]. Therefore, it is important to note that our predictive models are unlikely to account for 100% of the variability in patient outcomes that we observe. Instead, they will provide insight into the extent to which the presurgical biopsychosocial factors assessed in our model contribute to the observed variance.

### Future Directions and Concluding Comments

As per the data analysis plan for this study, a rigorous internal validation of the predictive models generated will be performed. Ongoing external validation of the models will also enable the suitability and specificity of the findings to be applied to a wider clinical setting than that included in this study. In addition, through future engagement with key stakeholders, including clinical care teams (surgical and rehabilitation specialists), health care providers, and patient advocates, we will develop the predictive models to optimize their clinical implementation and usefulness.

The classification models proposed will facilitate the evaluation of intervention studies, whereby biopsychosocial risk factors are identified, targeted interventions are provided, and the benefits to postoperative outcomes are evaluated. This will enable the determination of whether targeting these factors in an individualized manner leads to improved satisfaction and PROMs.

In conclusion, the findings of this study will contribute to the development of a comprehensive biopsychosocial clinical decision–predictive tool. Such a tool will have the potential to inform clinical care teams regarding individual patient outcomes, enable the stratification of potentially “at risk” individuals, and inform the evolution of rehabilitation services through the identification of potential interventions, all of which could lead to an improvement in patient outcomes and satisfaction following TKA.

## Abbreviations

BRS: Brief Resilience Scale
DASS: Depression Anxiety Stress Scales
MCID: minimal clinically important difference
NSW: New South Wales
PCS: Pain Catastrophizing Scale
PROM: patient-reported outcome measure
SF-12: Short Form-12
SSL: Secure Sockets Layer
TKA: total knee arthroplasty
VTOC: Vision Tree Optimal Care
WOMAC: Western Ontario and McMaster University Osteoarthritis Index
